# Adjuvante Systemtherapie beim Melanom in der Praxis: Multizentrische Erhebung an 51 DeCOG‐Hautkrebszentren

**DOI:** 10.1111/ddg.15963_g

**Published:** 2026-07-07

**Authors:** Markus Reitmajer, Elisabeth Livingstone, Lucie Heinzerling, Kai‐Martin Thoms, Frank Meiss, Markus V. Heppt, Anja Gesierich, Konstantin Drexler, Friedegund Meier, Max Schlaak, Andrea Forschner, Lisa Zimmer

**Affiliations:** ^1^ Universitäts‐Hautklinik Universitätsklinikum Tübingen; ^2^ Klinik für Dermatologie Universitätsklinikum Essen; ^3^ Klinik und Poliklinik für Dermatologie und Allergologie Ludwig‐Maximilians‐Universität München / Universitätsklinikum München; ^4^ Universitätsmedizin Göttingen; ^5^ Klinik für Dermatologie und Venerologie Universitätsklinikum Freiburg; ^6^ Hautklinik Universitätsklinikum Erlangen Friedrich‐Alexander‐Universität Erlangen‐Nürnberg (FAU), Erlangen; ^7^ Comprehensive Cancer Center Erlangen – Europäische Metropolregion Nürnberg (CCC ER‐EMN) Erlangen Deutschland; ^8^ Bayerisches Zentrum für Krebsforschung (BZKF) Universitätsklinikum Erlangen Friedrich‐Alexander‐Universität Erlangen‐Nürnberg (FAU), Erlangen; ^9^ Klinik für Dermatologie Universitätsklinikum Würzburg; ^10^ Klinik für Dermatologie Universitätsklinikum Regensburg; ^11^ Klinik für Dermatologie Universitätsklinikum Carl Gustav Carus Technische Universität Dresden; ^12^ Hautkrebszentrum am Universitätskrebszentrum Dresden und Nationales Centrum für Tumorerkrankungen, Dresden; ^13^ Klinik für Dermatologie Venerologie und Allergologie Charité – Universitätsmedizin Berlin

**Keywords:** Adjuvante Therapie, Immuncheckpoint‐Inhibitoren (ICI), Melanom, Nachsorge, PET/CT, Rezidiv, zielgerichtete Therapie (TT), Adjuvant therapy, follow‐up care, immune checkpoint inhibitor (ICI), PET/CT, recurrence, targeted therapy (TT)

## Abstract

**Hintergrund:**

Adjuvante Immuncheckpoint‐Inhibitoren (ICI) und zielgerichtete Therapien (*targeted therapy*, TT) haben das Therapiemanagement für Patienten mit Melanom grundlegend verändert. Die aktuelle deutsche S3‐Leitlinie unterscheidet jedoch nicht zwischen Patienten mit und ohne adjuvante Therapie in ihren Empfehlungen zu Bildgebungsintervallen und der Nachsorge nach Beendigung der adjuvanten Therapie. Dies führt zu uneinheitlichen Vorgehensweisen zwischen den Hautkrebszentren. Diese Studie gibt einen Überblick über die Vorgehensweise der Hautkrebszentren der Arbeitsgemeinschaft dermatologische Onkologie (ADO).

**Methodik:**

Am 22. November 2023 wurde eine Umfrage an 80 Hautkrebszentren in Deutschland, Österreich und der Schweiz versandt. Alle bis zum 10. März 2024 eingegangenen Antworten wurden deskriptiv analysiert.

**Ergebnisse:**

Die Rücklaufquote betrug 64% (n = 51). Vierzig Zentren (78%) wichen von der Leitlinie ab. In Stadium IIB führen 34 Zentren (67%) vor und nach adjuvanter Therapie CT‐Bildgebungen durch. Nach Abschluss der adjuvanten Therapie halten sich 36 Zentren (71%) an die Leitlinie. Über 90% bieten eine ICI an, wenn unter TT eine Progression auftritt – wie auch umgekehrt. Im Stadium IV würden 32 Zentren (63%) nach einem Rezidiv unter ICI eine TT anbieten.

**Schlussfolgerungen:**

Die Vorgehensweisen variieren und stimmen oft nicht mit der Leitlinie überein. Ein konsensbasierter Ansatz würde Patienten und Ärzten zugutekommen.

## EINLEITUNG

Die Zulassung von Immuncheckpoint‐Inhibitoren (ICI) und zielgerichteten Therapien (*targeted therapies*, TT) mit BRAF‐ und MEK‐Inhibitoren im Rahmen der Melanomerkrankung hat zu regelmäßigen Überarbeitungen der evidenzbasierten deutschen S3‐Leitlinie „Diagnose, Therapie und Nachsorge des Melanoms“ (Registrierungsnummer: 032/024OL) geführt, wobei die letzte Aktualisierung im Juli 2020 erfolgte.^1^

Im Jahr 2020 wurde die Empfehlung zur adjuvanten Therapie der Tumorstadien III–IV (AJCC 2017) mit Anti‐PD1‐Antikörpern und BRAF/MEK‐Inhibitoren als TT in die Leitlinie integriert.^1^, ^2^, ^3^, ^4^ Die Ergebnisse der Studien KEYNOTE‐716 und CheckMate 76K haben zur Zulassung von Pembrolizumab im Jahr 2022 und Nivolumab im Jahr 2023 für die adjuvante Behandlung des Melanoms im Stadium IIB und IIC geführt.^1^, ^5^, ^6^ Diese Empfehlungen sind in der aktuellen S3‐Leitlinie noch nicht integriert, werden aber im klinischen Alltag den Patienten als adjuvante Therapie angeboten. Neben den stadienabhängigen Nachsorgeempfehlungen der S3‐Leitlinien liegen keine gezielten Empfehlungen zur begleitenden Diagnostik, während und nach Abschluss der adjuvanten Systemtherapie, vor. Zum Beispiel wird im Stadium IIB nach den aktuellen Leitlinienempfehlungen keine radiologische Diagnostik durchgeführt. Insbesondere im Hinblick auf die frühzeitige Erkennung eines Fernrezidivs unter adjuvanter Therapie stellt sich die Frage, ob die Nachsorgeempfehlungen, einschließlich radiologischer Bildgebung und Labordiagnostik, für die Zeit vor, während und nach Abschluss der adjuvanten Therapie stadienübergreifend angepasst werden sollten – analog zu den entsprechenden adjuvanten Zulassungsstudien.^1^ So werden engere Intervalle als die sechsmonatlichen Abstände, die ab Stadium IIC für 3 Jahre empfohlen werden, und eine Ausweitung der radiologischen Bildgebung auch auf das Stadium IIB während der adjuvanten Therapiephase in Erwägung gezogen. Hintergrund dieser Überlegungen ist, dass die adjuvanten Therapien zum einen mit erheblichen Kosten verbunden sind und auf der anderen Seite auch ein hohes Nebenwirkungspotenzial haben. Es ist daher entscheidend, sicherzustellen, dass die Patienten tatsächlich davon profitieren. Die frühzeitige Erkennung von Metastasen ist im Blick auf Folgetherapien von entscheidender Bedeutung und Behandlungen, die sich als unwirksam, jedoch potenziell toxisch erweisen, sollten ohnehin frühestmöglich abgebrochen werden. Andererseits muss jedoch auch die erhöhte Strahlenexposition durch intensivere radiologische Bildgebungen, einschließlich der potenziellen Erhebung unspezifischer Befunde, sowie die psychologische Belastung der Patienten bis zur Befundmitteilung berücksichtigt werden.^7^, ^8^, ^9^, ^10^

Die Positronen‐Emissions‐Tomographie/Computertomographie (PET‐CT) hat sich als eine zuverlässige Methode für die primäre Bildgebung und Nachsorge etabliert. Darüber hinaus ist sie im Vergleich zur konventionellen Computertomographie (CT) hinsichtlich der Sensitivität überlegen.^11^, ^12^, ^13^, ^14^, ^15^ Was die landesweite Verfügbarkeit und Kostenübernahme des PET‐CT betrifft, so gibt es jedoch zwischen den Zentren deutliche Unterschiede, nicht nur innerhalb der Arbeitsgemeinschaft dermatologischer Onkologie (ADO).^13^ Die aktuellen Empfehlungen der deutschen S3‐Leitlinie beinhalten radiologische Bildgebung im Stadium IIC–IV alle 6 Monate während der ersten 3 Jahre. Im Stadium IIB wird lediglich eine Ultraschalldiagnostik empfohlen, ohne CT‐ oder MRT‐Untersuchungen. Es wird keine Unterscheidung gemacht, ob der Patient derzeit eine adjuvante Therapie erhält, diese bereits erhalten hat oder noch nie eine adjuvante Therapie hatte.^1^

Das *Survivorship‐Komitee* der ADO wurde im Juli 2023 gegründet und fördert einen offenen Austausch mit Patientenvertreterinnen und ‐vertretern.^16^ Es wurde dabei geäußert, dass Patienten wiederholt Unsicherheiten erleben, hinsichtlich der unterschiedlichen Handhabung von Bildgebungsintervallen und Diagnostikmodalitäten in den verschiedenen Hautkrebszentren während der adjuvanten Therapiephase sowie auch nach einer erneuten Progression. Dieser Apell wurde zum Anlass genommen, alle Hautkrebszentren innerhalb der ADO zu einer Umfrage einzuladen, um diese Beobachtung zu erfassen und gegebenenfalls zu objektivieren.

### Ziele

Ziel dieser Studie war es, das aktuelle Vorgehen bei der Behandlung von Patienten mit Melanom und adjuvanter Systemtherapie in den Hauttumorzentren der DeCOG in Deutschland, Österreich und der Schweiz zu erfassen und zu evaluieren, inwiefern die Zentren von den stadienabhängigen Nachsorgeempfehlungen der S3‐Leitlinie Melanom abweichen und eine intensivierte Diagnostik vor, unter und während adjuvanter Systemtherapie durchführen.

## MATERIAL AND METHODIK

### Studiendesign

Unter der Leitung des *Survivorship*‐Komitees der ADO wurde ein Fragebogen entwickelt (Tabelle S1 im Online‐Supplement). Der Fragebogen ist in zwei Abschnitte unterteilt: Der erste Teil konzentriert sich auf das diagnostische Vorgehen während und nach der adjuvanten Therapie, während der zweite Teil die Entscheidungsfindung im Falle einer Progression unter adjuvanter Therapie behandelt. Am 22. November 2023 wurde der Fragebogen an die Zentrumsleiter aller 80 zertifizierten Hautkrebszentren innerhalb der ADO verschickt, darunter 72 in Deutschland, vier in Österreich und vier in der Schweiz. Zwei Erinnerungen zur Teilnahme wurden versandt, am 11. Dezember 2023 und am 17. Januar 2024. Alle Antworten, die bis zum 10. März 2024 eingingen, wurden in die Auswertung einbezogen.

### Statistische Analyse

Der Fragebogen wurde in Microsoft^®^ Excel^®^ 2016 MSO (Version 2403) erstellt. Die deskriptive Analyse wurde mit IBM^®^ SPSS^®^ Statistics (Version 28.0.0.0) durchgeführt. Unvollständige Fragebögen wurden unter Verwendung der verfügbaren Antworten in die Auswertung einbezogen. Grafiken wurden mit GraphPad Prism^®^ (Version 10.0.1) erstellt.

## ERGEBNISSE

### Rücklaufquote des Fragebogens

Es sind 51 Antworten von 80 zertifizierten Hautkrebszentren in Deutschland, Österreich und der Schweiz eingegangen, was einer Rücklaufquote von 63,8% entspricht (Tabelle 1). Neunundvierzig Antworten kamen aus Deutschland, jeweils eine aus der Schweiz und aus Österreich. Mehr als die Hälfte der antwortenden zertifizierten Hautkrebszentren (n = 31, 60,8%) ist an Universitätskliniken angegliedert.

**TABELLE 1:** Zusammenfassung der Umfrageergebnisse von 51 Hautkrebszentren zur Stadieneinteilung während der adjuvanten Therapie.

	n	%
**Rücklaufquote des Fragebogens**		
Kontaktierte zertifizierte Hautkrebszentren innerhalb der ADO	80	100
Hautkrebszentren, die geantwortet haben (= Kohorte)	51/80	63,8
Keine Antwort	29/80	36,3
**Bildgebungen während der adjuvanten Therapie gemäß den Melanom‐Stadien in den Leitlinienempfehlungen**		
Ja	11/51	21,6
Individuelle Intervalle	40/51	78,4
**Bildgebung nach Beendigung der adjuvanten Therapie gemäß den Leitlinienempfehlungen**		
Ja	36/51	70,6
Individuelle Intervalle	15/51	29,4

### In den meisten Zentren wird ein engmaschigeres Monitoring als sechsmonatlich mit kürzeren Bildgebungsintervallen während der adjuvanten Therapiephase durchgeführt

**ABBILDUNG 1:**
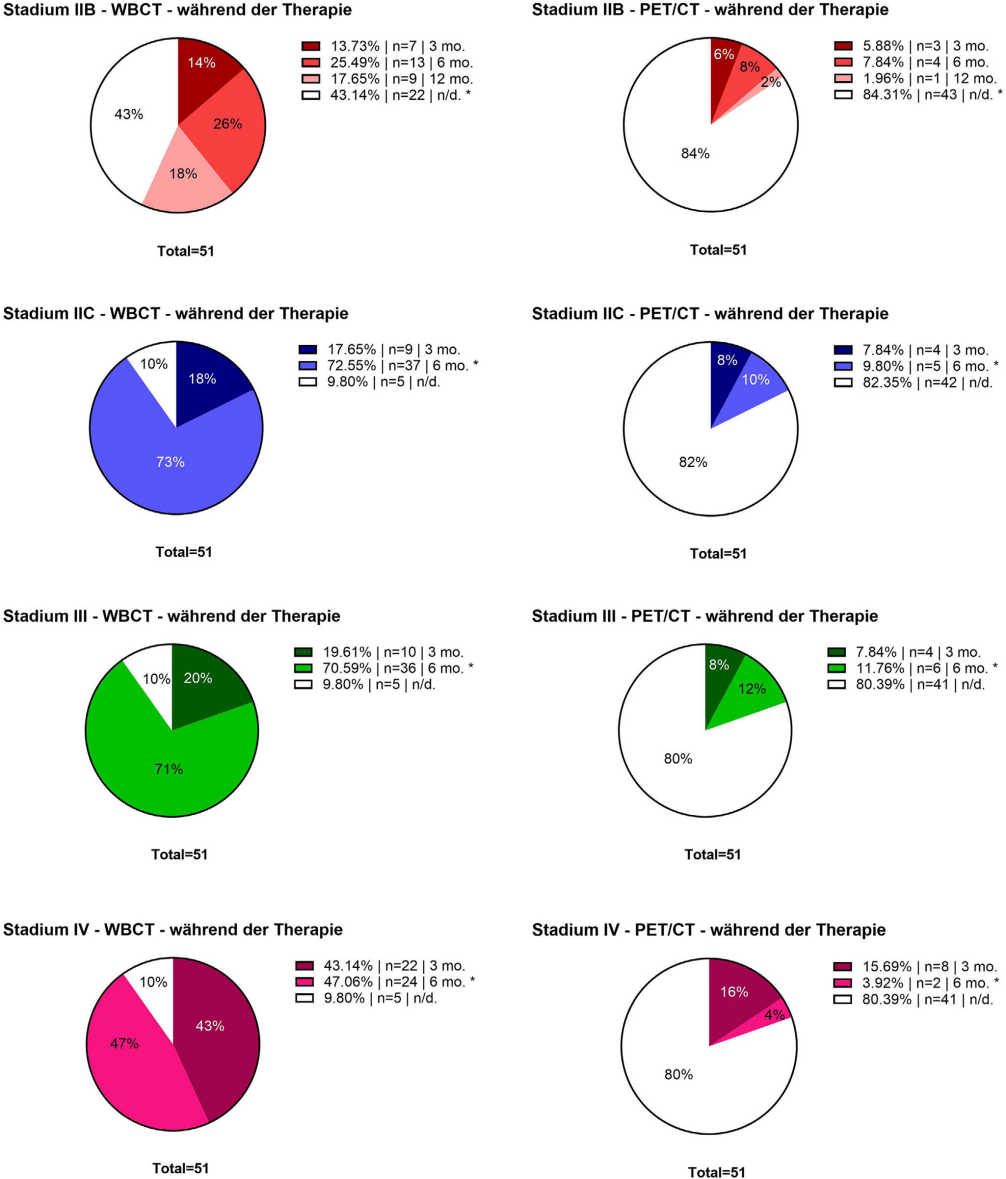
Bildgebungen und Intervalle während der adjuvanten ICI‐Therapie. Das empfohlene Intervall gemäß der aktuellen Leitlinie ist mit dem Symbol * gekennzeichnet. *Abk*.: mo, Monate; WBCT, *Whole‐Body*‐Computertomographie; WB PET/CT, *Whole‐Body*‐Positronen‐Emissions‐Tomographie/Computertomographie; n/d, nicht durchgeführt.

Bezüglich der Bildgebungsintervalle während der adjuvanten Therapie halten sich elf Zentren (22%) exakt an die in der aktuellen Leitlinie empfohlenen Intervalle (Tabelle 1). Das bedeutet, dass im Stadium IIB keine routinemäßige Bildgebung mittels CT oder Magnetresonanztomographie (MRT) durchgeführt wird und in den Stadien IIC–IV Ganzkörper‐CT (WBCT) sowie kraniale MRTs (cMRT)/kraniale CTs (cCT) in sechsmonatigen Abständen erfolgen. Die übrigen 40 Hautkrebszentren (78%) wichen von der Leitlinie ab und führten in den Stadien IIB–IV engmaschigere Bildgebungen durch (Abbildung 1, Tabelle 2).

**TABELLE 2:** Intervalle der durchgeführten Bildgebungen während und nach der adjuvanten Therapie im Detail.

Bildgebung vor Beginn der adjuvanten Therapie								
WBCT	Stadium IIB		Stadium IIC		Stadium III		Stadium IV	
	*n*	*%*	*n*	*%*	*n*	*%*	*n*	*%*
Ja, WBCT vor Beginn der adjuvanten Therapie	24	47,1	41	80,4	39	76,5	39	76,5
Ja, PET‐CT vor Beginn der adjuvanten Therapie	3	5,9	4	7,8	5	9,8	5	9,8
Ja, Bildgebung mit PET‐CT oder WBCT (je nach Situation) vor Beginn der adjuvanten Therapie	7	13,7	5	9,8	6	11,8	6	11,8
Nicht durchgeführt	17	33,3	–	–	–	–	–	–
Nicht beantwortet	–	–	1	2,0	1	2,0	1	2,0

Bildgebung während der adjuvanten Therapie								
WBCT	Stadium IIB		Stadium IIC		Stadium III		Stadium IV	
	*n*	*%*	*n*	*%*	*n*	*%*	*n*	*%*
3‐monatliches Intervall	7	13,7	9	17,6	10	19,6	22	43,1
6‐monatliches Intervall	13	25,5	37	72,5	36	70,6	24	47,1
12‐montliches Intervall	9	17,6	–	–	–	–	–	–
Nicht durchgeführt	22	43,1	5	9,8	5	9,8	5	9,8
Nicht beantwortet	–	–	–	–	–	–	–	–

Bildgebung während der adjuvanten Therapie								
WB PET/CT	Stadium IIB		Stadium IIC		Stadium III		Stadium IV	
	*n*	*%*	*n*	*%*	*n*	*%*	*n*	*%*
3‐monatliches Intervall	3	5,9	4	7,8	4	7,8	8	15,7
6‐monatliches Intervall	4	7,8	5	9,8	6	11,8	2	3,9
12‐montliches Intervall	1	2,0	–	–	–	–	–	–
Nicht durchgeführt	43	84,3	42	82,4	41	80,4	41	80,4
Nicht beantwortet	–	–	–	–	–	–	–	–

Bildgebung nach Beendigung der adjuvanten Therapie								
WBCT	Stadium IIB		Stadium IIC		Stadium III		Stadium IV	
	*n*	*%*	*n*	*%*	*n*	*%*	*n*	*%*
3‐monatliches Intervall	2	3,9	3	5,9	4	7,8	10	19,6
6‐monatliches Intervall	7	13,7	47	92,2	47	92,2	41	80,4
12‐montliches Intervall	–	–	–	–	–	–	–	–
Nicht durchgeführt	42	82,4	1	2,0	–	–	–	–
Nicht beantwortet	–	–	–	–	–	–	–	–

*Abk*.: mo, Monate; WBCT, *Whole‐Body*‐Computertomographie (Ganzkörper‐CT); WB PET/CT, *Whole‐Body*‐Positronen‐Emissions‐Tomographie/Computertomographie (Ganzkörper‐PET/CT); n/d, nicht durchgeführt; n, Anzahl

Alle Zentren (n = 50), die diesen Abschnitt des Fragebogens beantwortet haben, führen in den Stadien IIC, III und IV vor Beginn der adjuvanten Therapie bildgebende Untersuchungen durch. Im Stadium IIB führen 34 Zentren (67%) eine Bildgebung des gesamten Körpers zu Beginn und nach Beendigung der adjuvanten Therapie durch. Die Bildgebungsintervalle während der adjuvanten Therapie variieren zwischen den Zentren. Zwanzig Zentren (40%) führen zusätzlich auch während der adjuvanten Therapiephase Bildgebungen durch. In den Stadien IIC und III führen etwa 20% (9 Zentren im Stadium IIC und 10 Zentren im Stadium III) das *Staging* alle 3 Monate durch, anstatt des in der Leitlinie empfohlenen sechsmonatigen Intervalls für Patienten in den Stadien IIC–IV. Die Anzahl der Zentren, die ein verkürztes (3‐Monats‐)Intervall für das Monitoring verwenden, steigt im Stadium IV bei Patienten ohne Nachweis einer Erkrankung (NED) an. Im Stadium IV führen 22 Zentren (43%) eine Bildgebung alle 3 Monate durch. Ein PET‐CT wird von acht Zentren (16%) während der adjuvanten Therapie im Stadium IIB, von neun Zentren (18%) im Stadium IIC und von zehn Zentren (20%) in den Stadien III–IV eingesetzt (Tabelle 2).

### Die Bildgebung nach Beendigung der adjuvanten Therapie entspricht größtenteils den Empfehlungen der Leitlinie

**ABBILDUNG 2:**
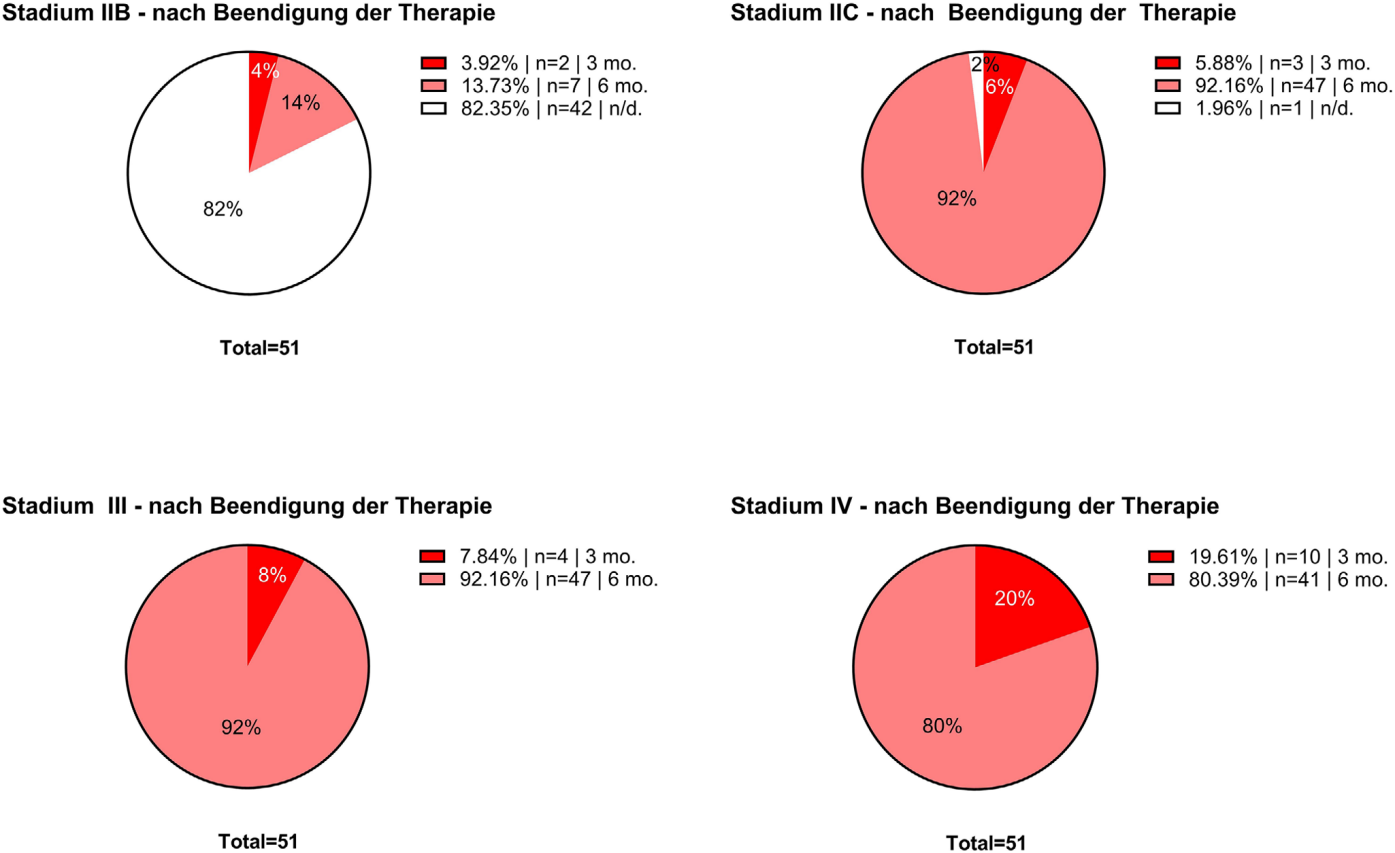
Bildgebungen und Intervalle nach Beendigung der adjuvanten ICI‐Therapie. *Abk*.: mo, Monate; n/d, nicht durchgeführt.

Hinsichtlich der Bildgebungsmodalität und der Frequenz der Intervalle nach Beendigung der adjuvanten Therapie folgen 36 Zentren (71%) den Empfehlungen der Leitlinie. Die aktuelle Leitlinie differenziert in ihrer Empfehlung nicht danach, ob eine adjuvante Therapie durchgeführt wird oder nicht. Die übrigen 15 Hautkrebszentren (29%) verkürzen die Intervalle überwiegend (Tabelle 2, Abbildung 2). Im Stadium IIB setzen neun Zentren (18%) die radiologische Bildgebung in drei‐ oder sechsmonatigen Intervallen nach Beendigung der adjuvanten Therapie fort. In den Stadien IIC und III halten sich 47 Zentren (92%) an die sechsmonatigen Intervalle. Im Stadium IV folgen 41 Zentren (80%) den sechsmonatigen Intervallen, während zehn Zentren (20%) kürzere Intervalle von 3 Monaten bevorzugen (Tabelle 2, Abbildung 2).

### Entscheidungsfindung im Falle eines Rezidivs während der adjuvanten Therapie

**ABBILDUNG 3:**
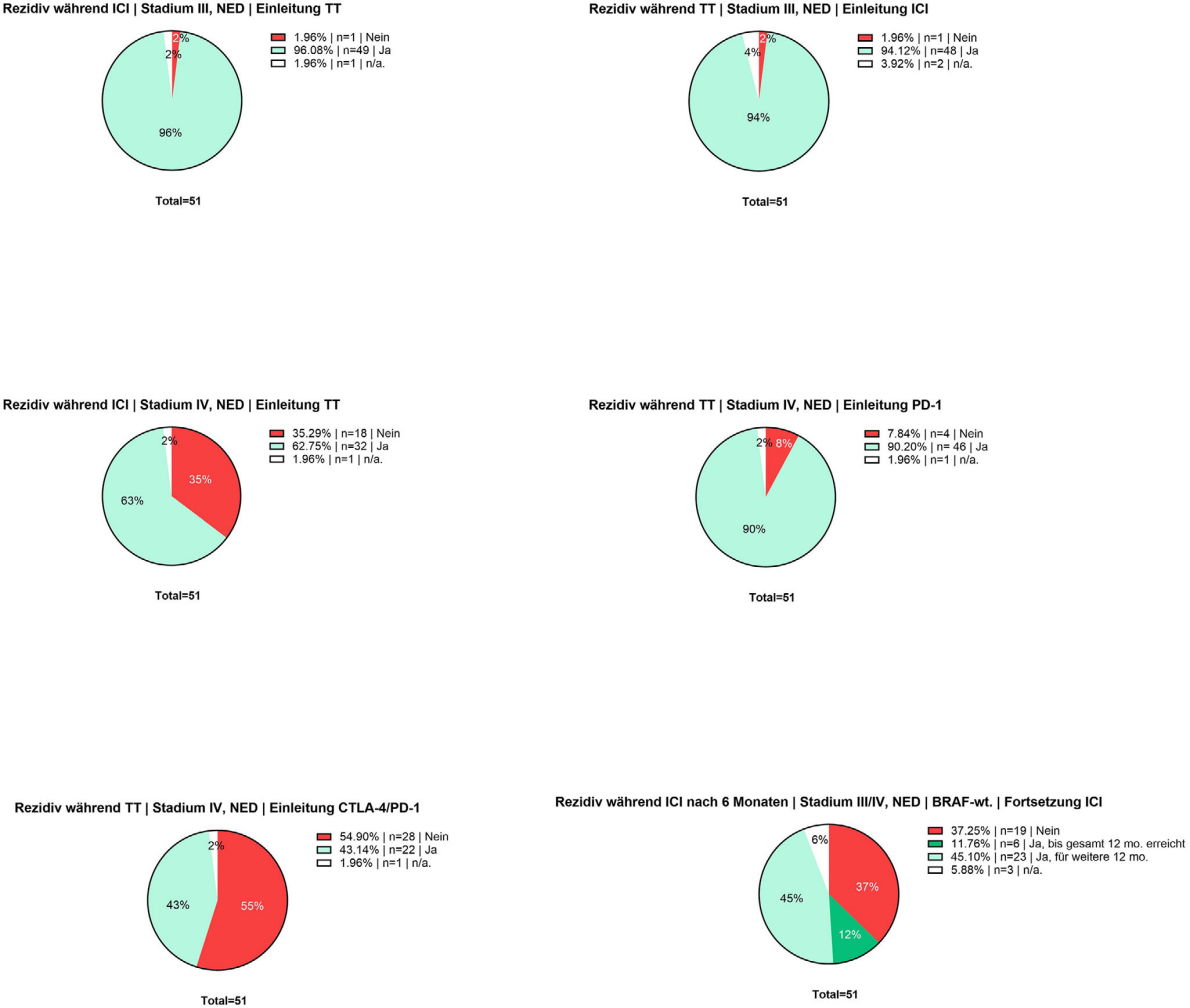
Entscheidungsfindung im Falle eines Rezidivs während der adjuvanten Therapie. *Abk*.: mo, Monate; n/a, keine Daten verfügbar; NED, keine Nachweise für eine Erkrankung; ICI, Immuncheckpoint‐Inhibitoren; TT, zielgerichtete Therapie.

Im zweiten Abschnitt des Fragebogens wurden typische klinische Situationen unter adjuvanter Therapie dargestellt, die eine Anpassung des ursprünglich geplanten Vorgehens erforderlich machten. Alle diese Szenarien beziehen sich auf die Entscheidungsfindung im Falle eines Rezidivs während der adjuvanten Therapiephase (Tabelle 3, Abbildung 3).

**TABELLE 3:** Entscheidungsfindung im Falle eines Rezidivs während der adjuvanten Therapie.

	n	%
**Rezidiv während ICI | Stadium III, NED | Einleitung TT**		
Nein	1/51	2,0
Ja	49/51	96,1
Keine Antwort	1/51	2,0
**Rezidiv während TT | Stadium III, NED | Einleitung ICI**		
Nein	1/51	2,0
Ja	48/51	94,1
Keine Antwort	2/51	4,0
**Rezidiv während ICI | Stadium IV, NED | Einleitung TT**		
Nein	18/51	35,3
Ja	32/51	62,7
Keine Antwort	1/51	2,0
**Rezidiv während TT | Stadium IV, NED | Einleitung PD‐1**		
Nein	4/51	7,8
Ja	46/51	90,2
Keine Antwort	1/51	2,0
**Rezidiv während TT | Stadium IV, NED | Einleitung CTLA‐4/PD‐1**		
Nein	28/51	54,9
Ja	22/51	43,1
Keine Antwort	1/51	2,0
**Rezidiv während ICI nach 6 Monaten | Stadium III/IV, NED | BRAF‐wt. | Fortsetzung ICI**		
Nein, keine Fortführung der Therapie	19/51	37,3
Ja, bis insgesamt 12 Monate erreicht worden sind.	6/51	11,8
Ja, für weitere 12 Monate	23/51	45,1
Keine Antwort	3/51	5,9

*Abk*.: ICI, Immuncheckpointinhibitor; TT, zielgerichtete Therapie (*targeted therapy*); PD‐1, *Programmed‐Death‐1*; CTLA‐4, *Cytotoxic‐T‐Lymphocyte‐Antigen‐4*; NED, *no evidence of disease* (kein Anhalt für Tumoraktivität); BRAF‐wt., BRAF‐Wildtyp; n, Anzahl

Nach vollständiger Resektion eines Rezidivs bei zuvor durchgeführter adjuvanter TT im Stadium III bieten 48 Zentren (94%) anschließend eine adjuvante ICI‐Therapie an. Umgekehrt berichteten 49 Zentren (96%), dass sie eine adjuvante TT nach vollständiger Resektion eines Rezidivs, das während einer adjuvanten ICI‐Behandlung im Stadium III bei Patienten mit BRAF‐V600‐Mutation festgestellt wurde, anbieten würden (Tabelle 3, Abbildung 3).

Im Falle eines Rezidivs mit resektablen Fernmetastasen im Stadium IV (NED) unter adjuvanter ICI‐Therapie, würden 32 Zentren (63%) nach erfolgreicher R0‐Resektion eine adjuvante TT anbieten (*Off‐Label*‐Gebrauch im Stadium IV, NED). Im Stadium IV und Rezidiv mit resezierbaren Metastasen während der adjuvanten TT, würden 46 Zentren (90%) nach erfolgter Resektion eine adjuvante ICI anbieten, während 22 Zentren (43%) auch die Option einer kombinierten ICI‐Therapie mit Ipilimumab/Nivolumab (*off label*) in Erwägung ziehen würden.

Im Fall resektabler Metastasen bei BRAF‐Wildtyp‐Situation und ≥ 6 Monaten adjuvanter ICI‐Therapie würden 19 Zentren (37%) die adjuvante ICI‐Therapie nach einer R0‐Resektion nicht fortsetzen. Sechs Zentren (12%) würden die ICI‐Therapie nach der Resektion für weitere 6 Monate fortsetzen, und 23 Zentren (45%) würden die adjuvante ICI‐Therapie für weitere 12 Monate nach der R0‐Resektion verlängern (Abbildung 3).

## DISKUSSION

Die neuen Therapieansätze mit ICI‐ und TT (BRAF/MEK‐Inhibitoren) haben das Vorgehen bei der Behandlung von Melanomen umfassend verändert. Mit ihrer Einführung in den adjuvanten Bereich und der mittlerweile erfolgten Zulassung auch für die Stadien IIB–IIC entstehen neue Fragestellungen und Anforderungen.^2^, ^3^, ^4^, ^5^ Aktuell wird die adjuvante Therapie mit verbesserter rückfallfreier Überlebenszeit und besserem fernmetastasenfreien Überleben assoziiert. Ein signifikanter Vorteil im Gesamtüberleben (*overall survival*, OS) konnte bisher jedoch nicht nachgewiesen werden – mit Ausnahme eines potenziellen OS‐Vorteils für Dabrafenib/Trametinib bei Patienten mit einem BRAF‐V600E‐mutierten Melanom.^2^, ^3^, ^17^, ^18^ Die *Number Needed to Treat* (NNT), um einen Rückfall bei Melanom im Stadium IIB/IIC zu verhindern, beträgt 7.81.^19^, ^20^ Aktuell variieren die internationalen Leitlinien hinsichtlich der Empfehlungen für die adjuvante Behandlung und Nachsorge bei fortgeschrittenem Melanom.^1^, ^21^, ^22^, ^23^, ^24^ Während die ASCO‐Leitlinie, der *ESMO*‐*Clinical‐Practice*‐Leitfaden und die NCCN‐Leitlinie (*National Comprehensive Cancer Network*) aktualisiert wurden und eine adjuvante Therapie mit Pembrolizumab oder Nivolumab für Patienten mit resektablem Melanom im Stadium IIB oder IIC des Melanoms empfehlen, ist dies in die aktuelle deutsche S3‐Leitlinie „Diagnostik, Therapie und Nachsorge des Melanoms“, die zuletzt 2020 aktualisiert wurde, aufgrund der erst erfolgten Zulassung der adjuvanten Therapie für das Stadium IIB–IIC im Jahr 2022/2023, noch nicht aufgenommen (Tabelle S2 im Online‐Supplement). Die *ESMO Clinical Practice Guideline 2025* betont, dass es keinen Konsens über den optimalen Nachsorgeplan oder den Nutzen bildgebender Verfahren bei reseziertem Melanom gibt. Sie empfiehlt daher, die jeweiligen nationalen Leitlinien zu konsultieren und diese entsprechend den verfügbaren Ressourcen anzupassen, insbesondere nach 3 Jahren Nachsorge. Im Gegensatz zur deutschen S3‐Leitlinie, die für Patienten im Stadium IIB keine routinemäßige Bildgebung vorsieht, empfiehlt die ESMO‐Leitlinie bereits in diesem Stadium ein *Staging* mittels Ultraschall, CT‐, PET‐Scans sowie eines MRI des Gehirns.^1^, ^25^ In allen genannten Leitlinien fehlen explizite Empfehlungen zu den Bildgebungsintervallen während und nach der adjuvanten Therapie, was zu unterschiedlichen Vorgehensweisen der Hautkrebszentren führt.^1^, ^23^, ^25^, ^26^, ^27^

In dieser multizentrischen Umfrage, die die Ergebnisse aus 51 Hautkrebszentren aus drei Ländern analysiert, wichen fast 80% der Zentren während der adjuvanten Therapiephase von der stadienabhängigen deutschen Leitlinienempfehlungen ab. Neunundzwanzig der 51 Zentren führen im Stadium IIB während der adjuvanten Therapie Bildgebungen in bis zu dreimonatigen Abständen durch. Derzeit sind keine Daten verfügbar, die sich mit der Frage befassen, inwiefern eine Bildgebung vor, während und nach der adjuvanten Therapie im Stadium IIB des Melanoms sinnvoll ist. Die Durchführung einer initialen Bildgebung und mindestens einer weiteren Bildgebung nach Abschluss der Therapie zielt darauf ab, einen Ausgangsbefund (inklusive eventueller pathologischer Veränderungen) vor Start vorliegen zu haben, und nach Abschluss der Therapie die Wirksamkeit und potenzielle Toxizitäten einer adjuvanten ICI besser bewerten zu können, um so eine erfolgreiche Nachsorge zu gewährleisten. So kann zum Beispiel ein Patient bereits vor Start einer adjuvanten Immuntherapie Lungengerüstveränderungen aufweisen, die bisher nicht bekannt sind und erst unter Immuntherapie zu klinischen Beschwerden führen. Ohne Ausgangsbefund ist unklar, ob diese Veränderungen bereits vorbestehend waren oder erst durch die Immuntherapie induziert wurden. Im Falle von bereits vorbesehenden Lungengerüstveränderungen, hätte eine Risiko‐Nutzen‐Abwägung hinsichtlich der adjuvanten Therapie gemeinsam mit dem Patienten besprochen werden können. Die Phase‐III‐Studien, die zur Zulassung der adjuvanten ICI führten, überprüften den Erfolg der Therapie alle 3–6 Monate bis zu 5 Jahre nach der Randomisierung in ihrem Studienprotokoll.^2^, ^3^, ^5^ Die CheckMate‐76K‐Studie und die KEYNOTE‐716‐Studie zeigten eine verbesserte Rate des rezidivfreien Überlebens (*recurrence‐free survival*, RFS) in der PD‐1‐Inhibitor‐Gruppe im Vergleich zur Placebo‐Referenzgruppe.^5^, ^6^ Dennoch wurde in der Pembrolizumab‐Gruppe (Stadium IIB) eine Rezidivrate von 20% nach 3 Jahren beobachtet, und in der Nivolumab‐Gruppe (Stadium IIB/C) von 22%.^20^, ^28^ Wenn man die gesamte Kohorte der Stadium‐IIB‐Patienten berücksichtigt, einschließlich derjenigen, die keine Therapie erhalten haben und daher nicht die Möglichkeit auf eine verbesserte RFS hatten, erscheint es sinnvoll, radiologische Bildgebung nicht nur bei Patienten durchzuführen, die eine adjuvante ICI‐Therapie erhalten, sondern auch bei denen, die diese nicht erhalten haben. Patienten ohne adjuvante ICI bleiben einem höheren Risiko für ein Rezidiv ausgesetzt, weshalb Bildgebung auch in dieser Gruppe von Nutzen sein kann, um ein frühzeitiges Wiederauftreten der Erkrankung zu erkennen. Im nicht‐adjuvanten Setting sprechen zwischen 40% und 60% der Patienten nicht auf eine ICI an. Wenn man hypothetisch annimmt, dass Patienten im adjuvanten Setting ähnlich wie im nicht‐adjuvanten Setting auf ICI ansprechen, könnte man schlussfolgern, dass etwa 50% der Patienten, die eine adjuvante ICI‐Therapie erhalten, nicht von der Behandlung profitieren.^29^, ^30^, ^31^ Eine regelmäßige Bildgebung könnte zudem zur frühzeitigen Erkennung und Therapie von Nebenwirkungen beitragen, wie zum Beispiel einer durch eine ICI assoziierte Pneumonitis.^32^, ^33^ Da auch spät auftretende immunbedingte Nebenwirkungen unter ICI‐Therapie vorkommen, stellt eine Behandlung trotz Progression ein unnötiges Risiko für Toxizität dar.^34^, ^35^ Eine der adjuvanten Systemtherapie angepasste intensivierte Bildgebung könnte daher das Nutzen‐Risiko‐Verhältnis verbessern.

Nach Beendigung der adjuvanten ICI‐Therapie im Stadium IIB führen neun Zentren auch im post‐adjuvanten Zeitraum weiterhin regelmäßig bildgebende Verfahren durch. Bei der Diskussion über die Notwendigkeit einer radiologischen Diagnostik im Stadium IIB ist jedoch zu berücksichtigen, dass eine erhöhte Strahlenbelastung potenzielle Konsequenzen mit sich bringt, einschließlich unspezifischer Befunde und der psychologischen Belastung für die Patienten, während sie auf die Ergebnisse der Untersuchung warten.^7^ Eine erhöhte Strahlenbelastung während der Nachsorge von Krebserkrankungen, insbesondere bei jungen Frauen, wird mit einem erhöhten Risiko für Brustkrebs im späteren Leben in Verbindung gebracht.^8^, ^9^, ^10^ Im Gegensatz zur aktuellen deutschen Leitlinie sind die Bildgebungsintervalle im Stadium IV (NED) in vielen Zentren häufiger als alle 6 Monate. Über 40% der Zentren führen während der adjuvanten Therapie alle 3 Monate eine Bildgebung mit WBCT durch, und auch nach Abschluss der adjuvanten Therapie setzt etwa ein Fünftel der Zentren diese in dreimonatigen Intervallen fort.

Im zweiten Teil des Fragebogens haben wir das Vorgehen bei einem Progress unter adjuvanter Therapie erfragt. Im Szenario eines BRAF‐mutierten Patienten mit lokoregionalem, aber resektablem Rezidiv (Stadium III) und adjuvanter ICI‐ oder TT‐Therapie würden mehr als 90% der Zentren nach vollständiger Entfernung des Rezidivs auf die andere adjuvante Therapie (ICI → TT / TT → ICI) wechseln. Im Gegensatz dazu unterschieden sich die Antworten hinsichtlich der anschließenden „zweiten adjuvanten“ Therapie bei Patienten im Stadium IV (NED) deutlich. Etwa 60% der Zentren würden nach Entfernung des Rezidivs *off‐label* eine adjuvante TT empfehlen, wenn zuvor eine adjuvante ICI‐Therapie durchgeführt wurde. Die Entscheidung der anderen Zentren, TT nicht anzubieten, kann auf die fehlende Zulassung von TT im adjuvanten Setting für Stadium‐IV‐Melanom (NED) sowie auf unzureichende Daten zu adjuvanter TT bei Patienten im Stadium IV zurückgeführt werden. Die aktuelle deutsche S3‐Leitlinie empfiehlt für Patienten mit Tumorstadium IV (NED) ausschließlich eine adjuvante ICI.^1^, ^36^ Retrospektive Daten deuten auf einen Vorteil in Bezug auf das rezidivfreie Überleben hin, jedoch auf Kosten von Toxizität, bei einer „zweiten adjuvanten“ Therapie mit TT nach Versagen der adjuvanten ICI‐Therapie bei Patienten im Stadium III.^37^, ^38^ Eine retrospektive multizentrische Analyse aus dem Hautkrebsregister ADOreg zeigte einen Nutzen der kombinierten adjuvanten ICI‐Therapie (anti‐PD1/anti‐CTLA4 ICI) im Falle eines Rezidivs nach adjuvanter TT.^37^ Die IMMUNED‐Studie zeigte, dass sowohl die Monotherapie mit anti‐PD1 als auch die kombinierte ICI‐Therapie das RFS bei Patienten mit Stadium IV Melanom und NED deutlich verbesserten, im Vergleich zur Placebogruppe. Das Gesamtüberleben war jedoch nur bei den Patienten besser, die die kombinierte ICI‐Therapie erhielten.^39^ Trotz dieser Daten bieten etwa 90% der Zentren nach der Resektion „nur“ eine PD‐1‐Monotherapie an, und weniger als die Hälfte der Zentren (43%) bietet eine adjuvante kombinierte ICI‐Therapie an, möglicherweise aufgrund der Einschränkung bei der Zulassung, die auf nicht resezierbare Erkrankungen beschränkt ist.

Insgesamt bietet diese multizentrische Studie wichtige Einblicke in die Praxis der Behandlung von Patienten mit Melanom und adjuvanter Systemtherapie. Das Vorgehen variiert zwischen den verschiedenen Zentren und es bestehen Diskrepanzen zwischen den Empfehlungen der aktuellen Leitlinie und der Praxis. In Anbetracht der hohen Kosten der adjuvanten Systemtherapie, der potenziell früher erkennbaren Nebenwirkungen in bildgebenden Verfahren, sowie der Bewertung des Nutzens der adjuvanten Therapie kann diskutiert werden, die Bildgebung mindestens zu Beginn, drei‐ oder sechsmonatlich während der adjuvanten Therapie und nach Beendigung auch bei Patienten im Stadium IIB durchzuführen.^32^, ^33^ Der Fragestellung, ob die Durchführung bildgebender Verfahren in der Nachsorge bei Hochrisiko‐Patienten die Gesamtüberlebensrate durch eine frühere Detektion verbessern kann, wird derzeit in der prospektiven, randomisierten, multizentrischen TRIM‐Studie (*Trial to Assess the Role of Imaging During Follow‐up After Radical Surgery of Stage IIB‐C and III Cutaneous Melanoma*, NCT 03116412) nachgegangen.^40^ Eine vorläufige Zwischenanalyse zeigte keinen Nutzen der Bildgebung im Nachsorgeprogramm für Hochrisiko‐Patienten.^41^ Allerdings haben bisher nur wenige Patienten die fünfjährige Nachbeobachtungszeit abgeschlossen. Der Studienabschluss wird für den 31. Dezember 2028 erwartet.^40^, ^41^ Die frühzeitige Erkennung eines Krankheitsrezidivs könnte jedoch eine frühere Einleitung nachfolgender Therapien bei vermutlich geringerer Tumorlast und niedrigeren LDH‐Werten ermöglichen. Dies könnte die Ergebnisse und das Überleben verbessern.^42^, ^43^, ^44^

Zwischen den Hautkrebszentren wurden relevante Unterschiede in der Bildgebung während der adjuvanten Therapie sowie in der Nachsorge nach Abschluss der Therapie festgestellt. Dies unterstreicht die Notwendigkeit eines standardisierten Vorgehens. Angesichts der raschen Entwicklungen und der zunehmenden Behandlungsoptionen bei der Diagnose eines fortgeschrittenen Melanoms besteht ein dringender Bedarf an kontinuierlichen Überarbeitungen der deutschen S3‐Leitlinie. Ein konsensbasiertes, einheitliches Vorgehen zwischen den Hautkrebszentren wäre sowohl für Patienten als auch für Ärzten von großem Vorteil.

## DANKSAGUNG

Wir möchten allen Hautkrebszentren für ihre Teilnahme an dieser Studie danken.

Open access Veröffentlichung ermöglicht und organisiert durch Projekt DEAL.

## FUNDING

M.R. erhielt Mittel im Rahmen des *Junior Clinician Scientists Program* der Universität Tübingen (Antragsnummer 523‐0‐0). Ansonsten hat diese Forschung keine spezifischen Fördermittel von öffentlichen, kommerziellen oder gemeinnützigen Organisationen erhalten.

## INTERESSENKONFLIKT

M.R. erhielt Reisekostenerstattungen von Almirall Hermal und Pierre Fabre außerhalb der eingereichten Arbeit. E.L. war als Beraterin tätig und hat Honorare von Bristol‐Myers Squibb, Merck Sharp & Dohme, Novartis, Pierre Fabre, Sanofi, Sun Pharma und Takeda erhalten. Außerdem erhielt sie Reisekostenerstattungen von Bristol‐Myers Squibb, Pierre Fabre, Sun Pharma und Novartis außerhalb der eingereichten Arbeit. L.H. war als bezahlte Beraterin für Bristol‐Myers Squibb, Immunocore, Novartis und Therakos tätig und ist an klinischen Studien innerhalb der Institution beteiligt (Agenus, Bristol‐Myers Squibb, Regeneron, Replimune, Huyabio International, Immunocore, IO Biotech, Merck Sharp & Dohme, Pfizer, Pierre Fabre, Sol‐Gel Technologies) außerhalb der eingereichten Arbeit. K.M.T. war als Berater tätig und hat Honorare für Vorträge von Bristol‐Myers Squibb, Merck Sharp & Dohme, Pierre Fabre, Novartis, Roche, Immunocore, Sanofi, Sun Pharma, Amgen, LEO, Galderma, Almirall, Candela und Lilly außerhalb der eingereichten Arbeit erhalten. Fra.M. hat Honorare von Novartis, Bristol‐Myers Squibb, Merck Sharp & Dohme, Pierre Fabre, Sanofi Genzyme und Sun Pharma erhalten. Darüber hinaus erhielt er Reisekostenerstattungen von Novartis, Sun Pharma, Pierre Fabre und Merck Sharp & Dohme außerhalb der eingereichten Arbeit. M.V.H. erhielt Honorare von Merck Sharp & Dohme, Bristol‐Myers Squibb, Roche, Novartis, Sun Pharma, Sanofi, Almirall, Biofrontera, Infectopharm, Immunocore und Galderma außerhalb der eingereichten Arbeit. A.G. war als Beraterin tätig und hat Honorare oder Reisekosten von Almirall, Amgen, Bristol‐Myers Squibb, Immunocore, Merck Sharp & Dohme, Novartis, Pierre Fabre Pharmaceuticals, Pfizer, Roche und Sanofi Genzyme erhalten außerhalb der eingereichten Arbeit. K.D. war als Berater tätig und hat Honorare von Pierre Fabre Pharmaceuticals, Novartis und Bristol‐Myers Squibb außerhalb der eingereichten Arbeit erhalten. Fri.M. hat Erstattungen von Reisekosten sowie Honorare für Beratung und Vorträge von Novartis, Roche, Bristol‐Myers Squibb, Merck Sharp & Dohme, Pierre Fabre, Sanofi und Immunocore erhalten sowie Forschungsunterstützung von Novartis und Roche außerhalb der eingereichten Arbeit. M.S. berichtet über Honorare für Vorträge und Advisory Boards sowie Reisekostenerstattungen von Bristol‐Myers Squibb, Merck Sharp & Dohme, Novartis, Pierre Fabre, Sun Pharma und Kyowa Kirin. A.F. war als Beraterin für Novartis, Merck Sharp & Dohme, Bristol‐Myers Squibb, Pierre Fabre und Immunocore tätig; sie erhielt Reisekostenerstattungen von Novartis, Bristol‐Myers Squibb und Pierre Fabre sowie Vergütungen für Vorträge von Novartis, Bristol‐Myers Squibb, Delcath und Merck Sharp & Dohme. Darüber hinaus erhielt sie Forschungsgelder von der Bristol‐Myers Squibb Stiftung Immunonkologie außerhalb der eingereichten Arbeit. L.Z. war als Beraterin tätig und hat Honorare von Bristol‐Myers Squibb, Merck Sharp & Dohme, Novartis, Pierre Fabre, Sanofi und Sun Pharma erhalten. Darüber hinaus erhielt sie Reisekostenerstattungen von Merck Sharp & Dohme, Bristol‐Myers Squibb, Pierre Fabre, Sanofi, Sun Pharma und Novartis außerhalb der eingereichten Arbeit.

## Supporting information



Supplementary information
