# Supplementary material for: Adjuvante Systemtherapie beim Melanom in der Praxis: Multizentrische Erhebung an 51 DeCOG‐Hautkrebszentren
Source: J Dtsch Dermatol Ges. 2026 Jul 7;24(7):916–27. [Article in German] doi: 10.1111/ddg.15963_g (PMC13340935; doi:10.1111/ddg.15963_g)
Supplement: Supplementary file 1 — Supplementary information [file DDG-24-916-s001.docx]

[[Supplements]]

**TABELLE S1** Fragebogen.

| **Wie führen Sie Ihre *Staging*-Untersuchungen bei adjuvanter Therapie durch?** | | | | | |
| --- | --- | --- | --- | --- | --- |
|  | | **Stadium IIB** | **Stadium IIC** | **Stadium III** | **Stadium IV** |
| ***Staging* bei adjuvanter Therapie** | Während adjuvanter Therapie nach Leitlinie (Stadium IIB ohne CT/MRT, IIC-IV alle 6 Monate WBCT, cMRI or cCT) |  |  |  |  |
|  | Während adjuvanter Therapie, Abweichung der Intervalle (dann Ausfüllen der nachfolgenden unten aufgelisteten Zeilen) |  |  |  |  |
| **CT Thorax, CT Abdomen, cMRI** | 1x vor Start der Therapie |  |  |  |  |
|  | alle 3 Monate unter Therapie |  |  |  |  |
|  | alle 6 Monate unter Therapie |  |  |  |  |
|  | 1x nach Abschluss der Therapie |  |  |  |  |
| **PET-CT mit cCT oder cMRI** | 1x vor Start der Therapie |  |  |  |  |
|  | alle 3 Monate unter Therapie |  |  |  |  |
|  | alle 6 Monate unter Therapie |  |  |  |  |
|  | 1x nach Abschluss der Therapie |  |  |  |  |
| ***Staging* nach Ende der adjuvanten Therapie** | Nach Abschluss der adjuvanten Therapie nach Leitlinie (Stadium IIB ohne CT/MRI, IIC-IV alle 6 Monate WBCT, cMRI or cCT für die ersten 3 Jahre) |  |  |  |  |
|  | Andere *Staging*-Intervalle 0 = keine *Stagings*, 1 = alle 3 Monate *Stagings* für 3 Jahre, Freitext |  |  |  |  |

| **Wenn bei Ihnen eine BRAF-mutierter Patientin/Patient einen lokoregionären Progress aber resektablen Progress unter adjuvanter Immuntherapie oder BRAF/MEK zeigt, bieten Sie ihm nach Resektion (und nachfolgender Tumorfreiheit Stadium III) dann die jeweils andere adjuvante Therapie an? (bitte entsprechendes ankreuzen)** | | |
| --- | --- | --- |
|  | **Ja** | **Nein** |
| d.h. bei Progress unter ICI, nach OP Angebot BRAF/MEK, wenn sich Pat. weiterhin im Stadium III NED befindet |  |  |
| d.h. bei Progress unter BRAF/MEK nach OP Angebot IO, wenn sich Pat. im Stadium III NED befindet |  |  |
|  | | |
| **Wenn bei Ihnen eine/ein BRAF-mutierter Patientin/Patient einen resektablen Progress mit Fernmetastase unter adjuvanter Immuntherapie oder BRAF/MEKi zeigt, bieten Sie ihm nach Resektion (und nachfolgender Tumorfreiheit im Stadium IV) dann die jeweils andere adjuvante Therapie an? (bitte entsprechendes ankreuzen)** | | |
|  | **Ja** | **Nein** |
| d.h. bei Progress unter ICI, nach OP Angebot BRAF/MEK, wenn sich Pat. im Stadium IV NED befindet |  |  |
| d.h. bei Progress unter BRAF/MEK nach OP Angebot ICI (PD1 Mono), wenn sich Pat. im Stadium IV NED befindet |  |  |
| d.h. bei Progress unter BRAF/MEK nach OP Angebot ICI (PD1 +CTLA4 Kombi), wenn sich Pat im Stadium IV NED befindet |  |  |
|  | | |
| **Wenn bei Ihnen eine/ein BRAF Wildtyp Patientin/Patient einen resektablen Progress unter adjuvanter Immuntherapie zeigt, nachdem er die adjuvante Immuntherapie bereits 6 Monate erhalten hat, bieten Sie ihm nach Resektion (und nachfolgender Tumorfreiheit Stadium III und IV) dann die Fortführung der adjuvanten Immuntherapie an?** | | |
|  | **Ja** | **Nein** |
| Ja, erneute Immuntherapie für 1 Jahr, gezählt ab dem Zeitpunkt der Resektion |  |  |
| Ja, Fortführung der Immuntherapie für insgesamt 1 Jahr, d.h. noch für weitere 6 Monate |  |  |
| Nein, Beendigung der Immuntherapie und Nachsorge nach Leitlinie |  |  |

**TABELLE S2** Vergleich der Leitlinien.

| Leitlinie | Empfehlung zur adjuvant PD-1 Therapie im Stadium IIB/C in der Leitlinie vorhanden? | Empfehlung zur adjuvanten PD-1 Therapie im Stadium III/IV in der Leitlinie vorhanden? | Empfehlung bezüglich der Bildgebungen während der adjuvanten Therapie in der Leitlinie vorhanden? | Stadium-abhängige Empfehlungen zur Bildgebung in der Leitlinie vorhanden? |
| --- | --- | --- | --- | --- |
| NCCN Guideline Melanoma: Cutaneous  Swetter et al. 2024 | Ja | Ja | Nein | Ja |
| Systemic Therapy for Melanoma: ASCO Guideline  Seth et al. 2023 | Ja | Ja | Nein | Nein |
| Dt. AWMF S3-Leitlinie Diagnostik,Therapie und Nachsorge des Melanoms AWMF [last update 2020] * | Nein | Ja | Nein | Ja |
| ESMO Clinical Practice Guidelines  Amaral et al. 2025 | Ja | Ja | Nein | Ja |

*Zulassung der adjuvanten Therapie erfolgte nach Veröffentlichung der Leitlinie.
